# Urine metabolome in women with *Chlamydia trachomatis* infection

**DOI:** 10.1371/journal.pone.0194827

**Published:** 2018-03-22

**Authors:** Claudio Foschi, Luca Laghi, Antonietta D’Antuono, Valeria Gaspari, Chenglin Zhu, Nicolò Dellarosa, Melissa Salvo, Antonella Marangoni

**Affiliations:** 1 Microbiology, DIMES, University of Bologna, Bologna, Italy; 2 Centre of Foodomics, Department of Agro-Food Science and Technology, University of Bologna, Cesena, Italy; 3 Dermatology, DIMES, University of Bologna, Bologna, Italy; UAMS/ACHRI/ACNC, UNITED STATES

## Abstract

The aim of this study was to characterize the urine metabolome of women with *Chlamydia trachomatis* (CT) uro-genital infection (n = 21), comparing it with a group of CT-negative subjects (n = 98). By means of a proton-based nuclear magnetic resonance (^1^H-NMR) spectroscopy, we detected and quantified the urine metabolites of a cohort of 119 pre-menopausal Caucasian women, attending a STI Outpatients Clinic in Italy. In case of a CT positive result, CT molecular genotyping was performed by *omp1* gene semi-nested PCR followed by RFLP analysis. We were able to identify several metabolites whose concentrations were significantly higher in the urine samples of CT-positive subjects, including sucrose, mannitol, pyruvate and lactate. In contrast, higher urinary levels of acetone represented the main feature of CT-negative women.

These results were not influenced by the age of patients nor by the CT serovars (D, E, F, G, K) responsible of the urethral infections. Since the presence of sugars can increase the stability of chlamydial proteins, higher levels of sucrose and mannitol in the urethral lumen, related to a higher sugar consumption, could have favoured CT infection acquisition or could have been of aid for the bacterial viability. Peculiar dietary habits of the subjects enrolled, in term of type and amount of food consumed, could probably explain these findings. Lactate and pyruvate could result from CT-induced immunopathology, as a product of the inflammatory microenvironment. Further studies are needed to understand the potential role of these metabolites in the pathogenesis of CT infection, as well as their diagnostic/prognostic use.

## Introduction

*Chlamydia trachomatis* (CT) represents the agent of the most common bacterial sexually transmitted infection (STI) worldwide, with more than 100 million new cases per year [1].

In women, uro-genital CT infections (i.e. urethritis, cervicitis) are often asymptomatic and, if left untreated, can lead to several complications and sequelae including pelvic inflammatory disease, tubal infertility and ectopic pregnancy [2].

CT is an obligate intracellular pathogen, showing a unique cycle of development with two distinct bacterial forms. The elementary bodies (EBs), infectious but non-dividing, enter epithelial cells and differentiate into reticulate bodies (RBs). After several rounds of replication by binary fission, RBs differentiate back into EBs and are released from the host cell 48–72 hours post-infection, ready to infect neighboring cells [3].

This development cycle requires a highly-regulated expression of chlamydial stage-specific genes and can be influenced by several environmental factors, as nutrient deprivation and exposure to host cytokines and antibiotics. In addition, chlamydiae modulate the host cell cycle progression and can cause substantial changes in gene expression and protein production in the host, at the transcriptional, translational and post-translational levels [3].

In this context, several in-vitro studies have investigated the metabolic adaptation of CT to mammalian host cells, as well as its stage-specific metabolic and transcriptional activity [4–6]. However, to the best of our knowledge, no data are available about the metabolic profile that can be found in human biological fluids during an ongoing CT infection.

The aim of this study was therefore to characterize the urine metabolome of women with CT uro-genital infection, comparing it with a group of CT-negative subjects. In particular, by means of a proton-based nuclear magnetic resonance (^1^H-NMR) spectroscopy, we detected and quantified the urine low weight metabolites of a cohort of 119 pre-menopausal Caucasian women, attending a STI Outpatients Clinic. ^1^H-NMR spectroscopy is a primary analytical technique used for metabolite detection, able to determine a molecular structure by measuring nuclear chemical shifts within a magnetic field [7]. In the recent past, such approach has been found useful to evidence consequences of inflammatory processes [8] and food habits [9, 10], thus appearing as promising in the present context.

## Materials and methods

### Study population and sample collection

From January 2016 to July 2016, all the pre-menopausal non-pregnant Caucasian women attending the STI Outpatients Clinic of Sant’Orsola-Malpighi Hospital in Bologna (Italy) and meeting one of the following criteria were enrolled: presence of uro-genital symptoms (i.e. vaginal discharge, dysuria, abnormal bleeding, dyspareunia) and/or presence of risk factors for CT infection (age < 25 years, new or multiple sexual partners, unsafe intercourses). Exclusion criteria comprised the use of any drugs or medications in the past month, the presence of chronic diseases and major endocrine or gynaecologic pathologies.

For all the patients, demographic data, behavioural characteristics and information about uro-genital symptoms were recorded. A written consent was obtained by all the patients and the study protocol was reviewed and approved by the Ethics committee of St. Orsola-Malpighi Hospital (7/2016/U/Tess).

Each enrolled subject provided the first void of the first urines of the morning. Then, after the interview and a clinical examination, a vaginal swab was collected by a clinician. The urine specimens were split within 3 hours of collection: 2.5 mL were used for CT, *Neisseria gonorrhoeae Trichomonas vaginalis* and *Mycoplasma genitalium* detection by commercial nucleic acid amplification techniques (NAATs) (Versant CT/GC DNA 1.0 Assay; Siemens Healthineers, Terrytown, NY, USA; Aptima *Trichomonas vaginalis* and Aptima *Mycoplasma genitalium* assay, Panther system, Hologic, Marlborough, MA, USA, respectively), whereas, 1 mL was frozen at –20°C. These specimens were kept frozen for a maximum of 1 month, prior to the ^1^H-NMR analysis and they were thawed only at the time of the metabolomic analysis, as described below.

The vaginal swab (E-swab, Copan, Brescia, Italy) was used for the molecular detection of CT, *N*. *gonorrhoeae*, *T*. *vaginalis* and *M*. *genitalium*, for the microscopic and culture-based diagnosis of candidiasis and aerobic vaginitis, as well as for bacterial vaginosis assessment (Nugent score > 7) [11].

Eligible women were allocated in one of the two following groups according on CT positivity (CT -positive group) or negativity (CT-negative group) in both the uro-genital tested sites.

### CT genotyping

In case of a CT positive result, the correspondent remaining eluate was recovered from Versant PCR plate and used for CT molecular genotyping [12]. Molecular genotyping was performed by *omp1* gene semi-nested PCR followed by RFLP analysis, as previously described [13, 14]. Briefly, the first product of 1033 base pairs (bp) was amplified using the following paired primers: SERO1A (5’-ATGAAAAAACTCTGAAATCGG-3’) and SERO2A (5’-TTTCTAGATCTTCATTCTTGTT-3’). Then, 1 μL of the first-round PCR product was used to amplify a 978 bp fragment, using the following primers: SERO2A and PCTM3 (5’-TCCTTGCAAGCTCTGCCTGTGGGGAATCCT-3’). After the PCR step, the amplified product was digested with AluI, DdeI and/or HinfI as restriction enzymes (Promega, Madison, USA) and visualized after electrophoresis run in ethidium bromide stained 12% polyacrylamide gel. CT serovar identification was achieved by the analysis of the specific restriction pattern.

### Urine metabolome analysis by ^1^H-NMR

**Fig 1:**
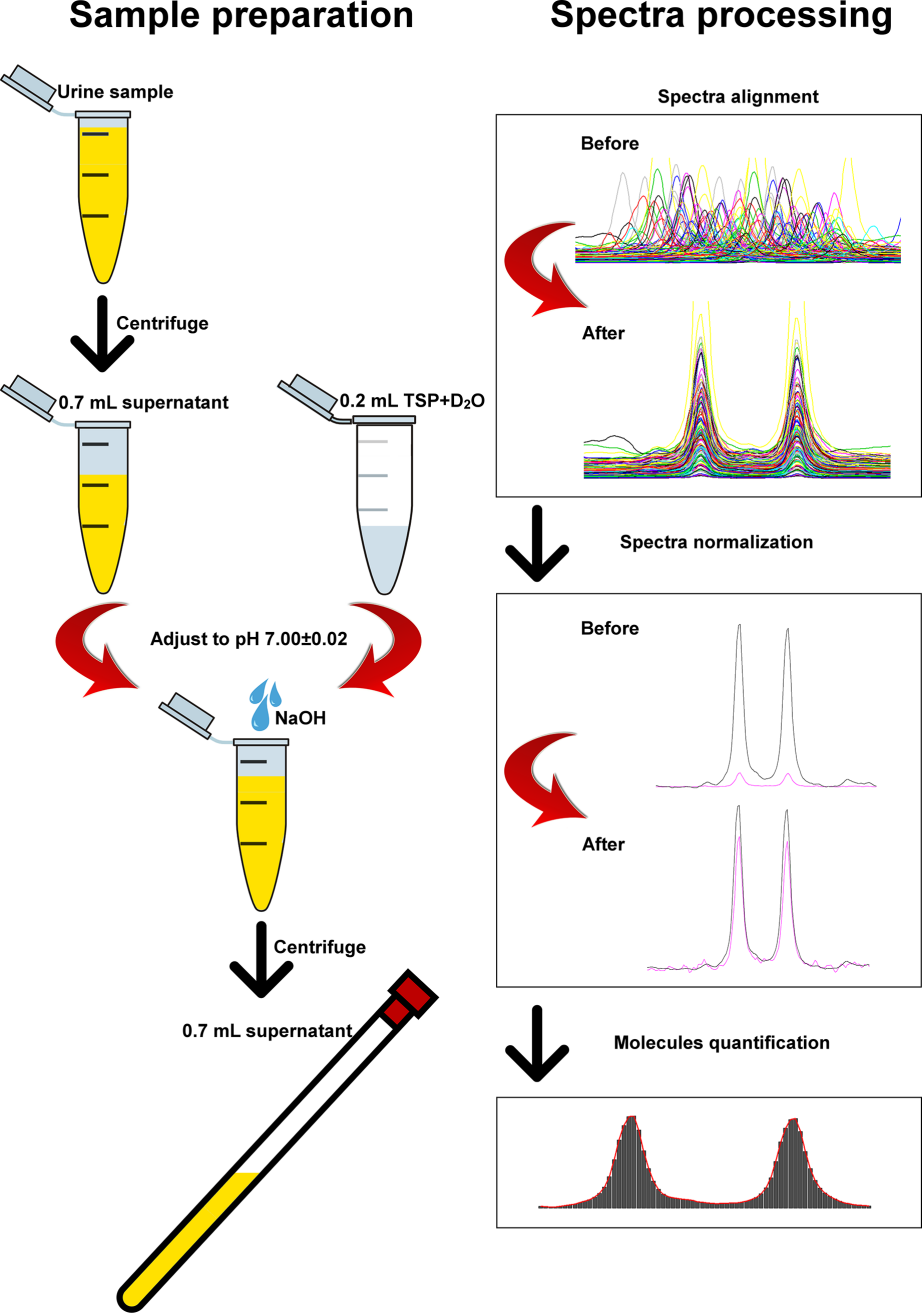
Graphic design of the workflow for urines preparation and ^1^H-NMR spectra processing. Urine samples were centrifuged and 700 μl of the supernatant were added to 0.2 mL of 3-(trimethylsilyl)-propionic-2,2,3,3-d4 acid (TSP) sodium salt 10 mM in deuterated water. The pH values of the solutions were adjusted to 7.0 by means of NaOH 1M and the samples were subjected to ^1^H-NMR spectroscopy. The spectra obtained by ^1^H-NMR were aligned and baseline-adjusted (normalization) and the signals were assigned to a specific metabolite by comparing their chemical shift and multiplicity with dedicated databases and libraries. Quantification of the molecules was achieved after the calculation of the area under each peak by means of a rectangular integration. The added TSP, at a known concentration, was employed as internal standard.

The general workflow of urine metabolome analysis by means of ^1^H-NMR is illustrated in Fig 1. Urine samples were prepared for ^1^H-NMR analysis by thawing them right before analysis. After centrifugation for 15 min at 18630 g at 4°C, an amount of supernatant equal to 700 μL was added to 200 μL of a D_2_O solution of 3-(trimethylsilyl)-propionic-2,2,3,3-d4 acid (TSP) sodium salt 10 mM, buffered at pH 7.00 ± 0.02 by means of 1M phosphate buffer. The pH of each sample was then finely adjusted to pH 7.00 ± 0.02 by the addition of a few drops of NaOH (1 M). ^1^H-NMR spectra were recorded at 298 K with an AVANCE III spectrometer (Bruker, Milan, Italy) operating at a frequency of 600.13 MHz, equipped with Topspin software (Ver. 3.5).

The residual water signal was suppressed by applying the first increment of the nuclear Over-hauser effect spectroscopy (NOESY) pulse sequence and a spoil gradient. This was done by employing the NOESYGPPR1D sequence, part of the standard pulse sequence library. Each spectrum was acquired using 32,000 data points over a 7,211.54 Hz spectral width and adding 256 transients. A recycle delay of 5 s and a 90° pulse of 11.4 μs were set up.

Acquisition time (2.28 s) and recycle delay were adjusted to be 5 times longer than the longitudinal relaxation time of the protons under investigation, which was considered to be not longer than 1.4 s.

To each spectrum, line broadening (0.3 Hz) and phase adjustment was applied by Topspin software, while any further spectra processing, molecules quantification and data mining step was performed in R computational language (R: A Language and Environment for Statistical Computing) by means of scripts developed in house.

The spectra were aligned towards the right peak of alanine doublet, set to 1.473 ppm, and the signals of water and urea were removed. The spectra were then baseline-adjusted by means of peak detection according to the “rolling ball” principle [15] implemented in the “baseline” R package [16]. A linear correction was then applied to each spectrum, so to make the points pertaining to the baseline randomly spread around zero. No manual alignment of the signals was necessary, differently from previous investigations [8].

The signals were assigned by comparing their chemical shift and multiplicity with the Human Metabolome Database [17] and the compounds library (Ver. 10) of Chenomx software (Chenomx Inc., Canada, Ver. 8.3). Quantification of the molecules was performed in the first sample acquired by employing the added TSP as an internal standard. In order to compensate for differences in solids content, any other sample was then normalized to such sample by means of probabilistic quotient normalization [18], set up so to exclude spectra portions ascribed to water and urea. Integration of the signals was performed for each molecule by means of rectangular integration.

### Statistical analysis

All statistical analysis were performed by using R computational language. In order to evaluate statistically significant differences between the two groups of subjects, Wilcoxon test was used to compare quantitative data, while categorical data were analyzed with Fisher’s exact test. Differences in urine metabolites between groups were calculated by means of a two-tailed unpaired Wilcoxon test. Interactions among variables (i.e. the age of patients and metabolite concentrations) on metabolome features were analyzed, by expressing the concentration of each molecules as ranks, followed by two-way ANOVA, as previously suggested [19].

In order to summarize the information from univariate analysis, robust principal component analysis (rPCA) [20] has been chosen as the election chemometric technique. Principal component analysis algorithm rotates the original space represented by the concentration of each molecule, so to show the samples from the point of view representing the greatest percentage of the samples’ variance. The scoreplot, the representation of the samples from the new point of view, helps to visually inspect the trends underlying the samples, while the loadingplot (barplot) evidences the molecules mainly driving the trends. Statistical significance was determined at *P* < 0.05.

## Results

### Study group

During the study period, a total of 119 subjects met the inclusion criteria and were enrolled. In particular, 98 women were allocated in the CT-negative group, whereas 21 showed a contemporary positivity for CT both on urines and on the vaginal swab and were assigned to the CT-positive group. Clinical, behavioural and demographic information of the study groups are reported in details in Table 1.

**Table 1:** Demographic, behavioural and clinic characteristics of the women enrolled for the study.

	CT negative	CT positive	*P* value
	(n = 98)	(n = 21)	
Mean age ± SD (years)	28.6 ± 7.4	24.3 ± 3.6	0.01
Sexual orientation			
-heterosexual	98/98 (100%)	21/21 (100%)	-
-homosexual	0/98 (0%)	0/21 (100%)	-
Previous STIs			
-syphilis	12/98 (12.2%)	3/21 (14.2%)	0.72
-urogenital CT infection	17/98 (17.3%)	4/21 (19.0%)	1.0
-genital HPV	7/98 (7.1%)	1/21 (4.8%)	1.0
Partner positive for CT	2/98 (2.1%)	5/21 (23.8%)	0.002
Symptoms			
-genital symptoms	59/98 (60.2%)	9/21 (42.8%)	0.15
-urethral symptoms	11/98 (11.2%)	2/21 (9.5%)	1.0

Notably, more than half (12/21; 57.1%) of CT-positive patients were completely asymptomatic. In case of symptoms, they complained about the presence of dyspareunia (6/9) and vaginal discharge (5/9) and less frequently about abnormal bleeding (3/9) and dysuria (2/9).

The cohort of the 98 women of the CT-negative group included 20 patients with bacterial vaginosis, 14 subjects with vulvo-vaginal candidiasis, 3 women with *Mycoplasma genitalium* infection, 1 case of trichomoniasis and 60 women (50% with various uro-genital symptoms) negative for all the microbiological tests performed. Patients with *T*. *vaginalis* and *M*. *genitalium* infections were found positive only at the vaginal level, thus excluding an urethral involvement.

### CT genotyping

The most common CT serovar in our population was E (11/21; 52.4%) followed by F (3/21; 14.3%), G (3/21; 14.3%), D (2/21; 9.5%) and K (2/21; 9.5%). No significant associations between CT serovar and the presence of symptoms were found.

### Metabolome analysis

The urine samples were subjected to ^1^H-NMR spectroscopy in order to determine the metabolic profiles in the different groups. By means of the metabolomic analysis, we detected and quantified 66 molecules, mainly belonging to the groups of sugars, organic acids, nitrogen compounds and amino acids (S1 Table). An example of a visual representation of the molecules assignment and quantification procedure is depicted in S1 Fig.

By means of univariate analysis, differences in the metabolic profiles were searched between CT-positive and CT-negative groups. Table 2 lists the metabolites whose concentration significantly differed between the two. In particular, higher concentrations of 2-furoylglycine, sucrose, threonine, methylsuccinate, lactate, mannitol and pyruvate were found in CT-positive subjects, whereas CT-negative patients were characterized by higher levels of acetone, hypoxanthine and methylguanidine. The range of concentrations of these metabolites were in line with previous reports, evaluating the urinary levels in healthy adults (S2 Table). These data can be found on the ‘human metabolome database’ (HMDB; www.hmdb.ca), a freely available electronic database containing detailed information about small molecule metabolites found in the human body.

**Table 2:** Molecules whose concentration (mM, mean ± SD) showed significant differences (*P*<0.05) in relation to *Chlamydia trachomatis* positivity. CT-: *C*. *trachomatis*-negative; CT+: *C*. *trachomatis*-positive.

	CT-	CT+	*P*
Hypoxanthine	1.48E-1 ± 1.91E-1	8.92E-2 ± 4.97E-2	0.01
2-Furoylglycine	9.08E-2 ± 1.05E-1	1.39E-1 ± 1.19E-1	0.03
Sucrose	6.79E-2 ± 5.63E-2	2.37E-1 ± 6.46E-2	< 0.0001
Threonine	2.04E-1 ± 7.22E-2	2.38E-1 ± 6.47E-2	0.03
Lactate	3.02E-1 ± 3.82E-1	3.48E-1 ± 1.20E-1	0.002
Mannitol	2.20 ± 2.66	2.65 ± 1.72	0.003
Methylguanidine	4.66E-2 ± 2.57E-2	3.84E-2 ± 3.18E-2	0.01
Pyruvate	1.91E-2 ± 9.07E-3	3.62E-2 ± 5.89E-2	0.02
Acetone	4.78E-2 ± 2.59E-1	9.86E-3 ± 3.34E-3	0.01
Methylsuccinate	5.99E-2 ± 4.04E-2	7.80E-2 ± 3.56E-2	0.004

Considering that CT-infected women were significantly younger than CT-negative ones, we wondered if the age of the patients was a confounding factor on the effects of chlamydia on metabolomic profiles. By means of a two way ANOVA, we excluded that age exerted any influence on the urine metabolome of the two study groups.

**Fig 2:**
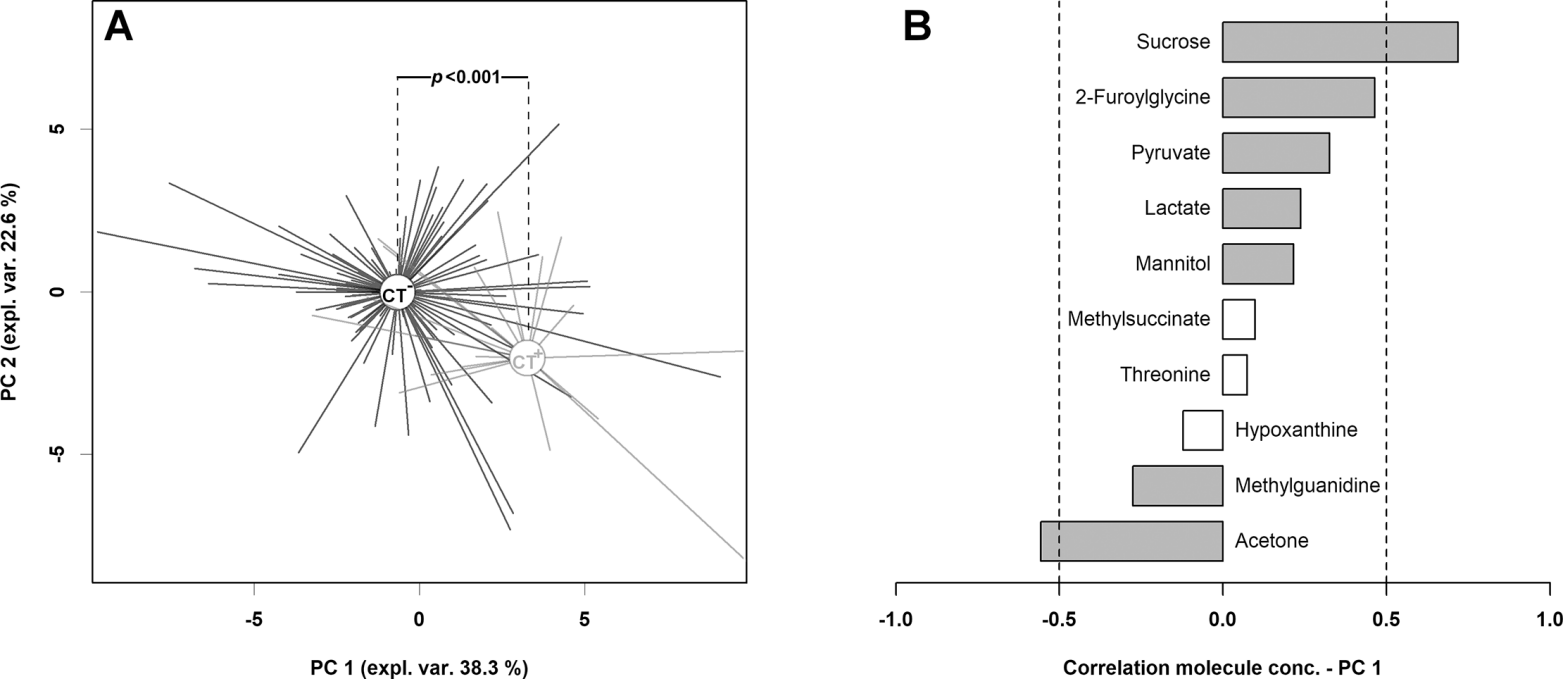
rPCA model calculated on the space constituted by the concentration of the molecules listed in Table 1. In the scoreplot (A), CT-negative (CT-) and CT-positive (CT+) subjects are represented in black and gray respectively, with lines connecting each subject to the median of its group. In the barplot (B), describing the correlation between the concentration of each molecule and its importance over PC 1, dark gray bars highlight statistically significant correlations (*P*<0.05).

In order to highlight underlying trends in the metabolome subspace represented by these molecules, their concentration was employed as a base for the calculation of a robust principal component analysis (rPCA) model (Fig 2).

Six components were found to describe robustly the samples. PC1, explaining the 38.3% of the total variance, effectively accounted for the presence of *C*. *trachomatis* infection, with CT-negative subjects appearing at low PC1 scores and CT-positive ones characterized by high value scores (*P*<0.001). The correlation between the concentration of each molecule and its importance over PC1 served as a handy way to rank the molecules in order of importance in determining such grouping, with high concentrations of sucrose and low concentrations of acetone representing the main features of *C*. *trachomatis* presence.

The composition of urine metabolites did not vary significantly between the different CT serovars responsible for the infections.

## Discussion

In this study we characterized the metabolic profile of urine samples of two groups of pre-menopausal women: 21 patients with uro-genital *C*. *trachomatis* infections and 98 CT-negative subjects. The relevance of a deep comprehension of the features of the urine metabolome in CT-positive women lies in several aspects: (i) getting new insights in the pathogenesis of CT urethral infections; (ii) identifying metabolites that may favor CT infection acquisition or persistence; (iii) opening the perspective of new diagnostic approaches based on the detection and quantization of urine metabolites.

By means of a ^1^H-NMR analysis, we were able to identify several metabolites whose concentrations were significantly higher in the urine samples of CT-positive subjects, including sucrose, mannitol, pyruvate and lactate. These results were comparable for all the CT serovars detected (D, E, F, G, K), thus indicating a common behaviour in presence of chlamydia, irrespective of the specific serovars.

At first, we hypothesized that a higher level of sucrose in the urethral lumen, related to a higher sugar consumption, could have favored CT acquisition or delayed its clearance. Indeed, it has been shown that urinary sucrose is a biomarker for total sugar consumption, considering that the diet-intake of sucrose is significantly correlated with its concentration in urines [21, 22]. In this context, it has been demonstrated that sucrose can increase the stability of chlamydial proteins (i.e. MOMP: major outer membrane proteins), thus potentially leading to a longer viability of CT elementary bodies [23].

In a similar way, mannitol, a sugar alcohol often found in hard candies, fruits and vegetables, could act as a ‘stabilizer’ of proteins and membranes, lengthening the bacterial viability [24–26].

Second, we highlighted a significant difference in the concentration of 2-furoylglycine and acetone between the study groups. It has been shown that 2-furoylglycine at the urinary level can be considered as a biomarker of caffeine consumption [27] and, in addition, can derive from furan derivatives found in food prepared by strong heating [28]. Contrariwise, the urinary excretion rate of acetone, one of the ketone bodies, is found to be higher during fasting or low-sugar diet [29].

Peculiar dietary habits of the subjects enrolled, in term of type and amount of food consumed and presence of fasting periods, could probably explain these findings.

Finally, we supposed that the presence of higher levels of pyruvate and lactate in urines of infected women could be ascribed to CT-induced immunopathology. Following CT uro-genital infection, an early acute inflammatory response occurs, characterized by secretion of pro-inflammatory cytokines (TNF-α, IL-8, IL-1, GM-CSF) and recruitment of immune cells (macrophages, CD4+ and CD8+ T cells, neutrophils) [30, 31]. A key feature of the inflammatory microenvironment is the accumulation of lactate, a by-product of highly proliferating cells during the glycolytic pathway [32, 33]. Indeed, it has been shown that, at the inflammatory sites, a metabolic switch to aerobic glycolysis occurs in T lymphocytes. This change leads to the up-regulation of glycolytic enzymes and glucose transporters to the membrane, with an increase in glycolytic flux and the concomitant production of lactate, derived from the conversion of pyruvate [32–34]. In this context metabolomics has been recently and successfully employed to show that the accumulation of lactate is a marker of inflammation in several organs and tissues [35–38].

We are fully aware that some limitations could have affected our study. At first, the lack of information about the type and amount of leukocytes in the urethral fluids of patients made any association between metabolic changes and inflammation impossible. Second, the availability of data about dietary habits or food consumption would have strengthened the metabolomic results obtained. Even though the data presented here are mainly preliminary and descriptive, this study can open the way to the use of new ‘omic’ sciences, as metabolomics, to deepen host-pathogen interactions and to better understand *C*. *trachomatis* pathogenesis.

## Conclusions

To the best of our knowledge this is the first report investigating the urine metabolome in women with CT uro-genital infection. We were able to identify several metabolites whose concentrations were significantly higher in the urine samples of CT-infected women. Further studies are needed to understand the accurate origin of the urine metabolites and to comprehend if the observed alterations precede or follow the infection onset. Moreover, the potential role of these metabolites in the pathogenesis of CT infection, as well as their diagnostic/prognostic use, should be investigated.

## Supporting information

S1 Fig Visual representation of the molecules assignment and quantification procedure by means of Chenomx software (Chenomx Inc., Canada, Ver. 8.3) and R script developed in house. Above panel—portions of the spectra, superimposed in white-washed mode. The black and red dashed lines show the portions of the spectra employed for the quantification of each molecule.
Below panel–one representative spectrum (black line) superimposed to the signals simulated by software Chenomx (red line) for each of the molecules listed. The line connecting each name with the spectra highlight the signal used for quantification purposes. The quantification of the molecules was performed in the first sample acquired by employing the added TSP (3-(trimethylsilyl)-propionic-2,2,3,3-d4 acid sodium salt), at a known concentration, as internal standard. The area under the peak of each molecule was calculated after the integration of the signals by means of a rectangular integration.
(DOCX)

S1 Table Concentration (mM) of urine metabolites determined by ^1^H-NMR. Results are expressed as mean ± standard deviation. CT+: *Chlamydia trachomatis*-positive women; CT-: *Chlamydia trachomatis*-negative women.
(DOCX)

S2 Table List of references used to compare the range of concentrations of urine metabolites found in this study with previous works on healthy adults. These data can be found on the ‘human metabolome database’ (HMDB; www.hmdb.ca), a freely available electronic database containing detailed information about small molecule metabolites found in the human body.
(DOCX)
