# Supplementary material for: Urine metabolome in women with Chlamydia trachomatis infection
Source: PLoS One. 2018 Mar 22;13(3):e0194827. doi: 10.1371/journal.pone.0194827 (PMC5864028; doi:10.1371/journal.pone.0194827)
Supplement: S1 Table — Results are expressed as mean ± standard deviation. CT+: Chlamydia trachomatis-positive women; CT-: Chlamydia trachomatis-negative women. (DOCX) [file pone.0194827.s002.docx]

| **Metabolites** | **CT- (n=98)** | **CT+ (n=21)** |
| --- | --- | --- |
| 1-methylnicotinamide | 0.0203 ± 0.013 | 0.0209 ± 0.016 |
| formate | 0.113 ± 0.05 | 0.111 ± 0.04 |
| hypoxanthine | 0.147 ± 0.19 | 0.089 ± 0.04 |
| 4-hydroxybenzoate | 0.054 ± 0.05 | 0.052 ± 0.04 |
| hippurate | 2.356 ± 1.99 | 2.293 ± 1.54 |
| 3-indoxylsulfate | 0.118 ± 0.05 | 0.133 ± 0.05 |
| N-phenylacetylglycine | 0.389 ± 0.18 | 0.402 ± 0.15 |
| 3-hydroxymandelate | 0.273 ± 0.18 | 0.264 ± 0.17 |
| tyrosine | 0.044 ± 0.01 | 0.048 ± 0.02 |
| 2-furoilglycine | 0.090 ± 0.10 | 0.139 ± 0.11 |
| fumarate | 0.002 ± 0.00 | 0.003 ± 0.00 |
| orotic acid | 0.001 ± 0.00 | 0.001 ± 0.00 |
| ADP | 0.010 ± 0.00 | 0.011 ± 0.00 |
| sucrose | 0.067 ± 0.05 | 0.237 ± 0.06 |
| allantoin | 0.053 ± 0.02 | 0.066 ± 0.03 |
| xylose | 0.016 ± 0.02 | 0.017 ± 0.01 |
| fucose | 0.029 ± 0.01 | 0.031 ± 0.01 |
| arabinose | 0.156 ± 0.38 | 0.172 ± 0.38 |
| trigonelline | 0.131 ± 0.09 | 0.143 ± 0.09 |
| hydroxyacetone | 0.077 ± 0.07 | 0.096 ± 0.07 |
| tartrate | 0.030 ± 0.03 | 0.039 ± 0.03 |
| threonine | 0.204 ± 0.07 | 0.237 ± 0.06 |
| lactate | 0.302 ± 0.38 | 0.348 ± 0.11 |
| creatine | 0.291 ± 0.36 | 0.279 ± 0.19 |
| mannitol | 2.200 ± 2.66 | 2.645 ± 1.72 |
| theophylline | 0.020 ± 0.01 | 0.029 ± 0.02 |
| glycine | 1.652 ± 0.92 | 1.548 ± 0.86 |
| trans-aconitate | 0.122 ± 0.06 | 0.127 ± 0.06 |
| taurine | 0.783 ± 0.58 | 0.514 ± 0.28 |
| methanol | 0.117 ± 0.07 | 0.109 ± 0.05 |
| TMAo | 0.245 ± 0.30 | 0.145 ± 0.04 |
| betaine | 0.055 ± 0.03 | 0.044 ± 0.01 |
| carnitine | 0.045 ± 0.04 | 0.060 ± 0.05 |
| N-nitrosodimethylamine | 0.020 ± 0.00 | 0.019 ± 0.00 |
| dimethylsulfone | 0.043 ± 0.01 | 0.045 ± 0.01 |
| malonate | 0.047 ± 0.01 | 0.052 ± 0.01 |
| cis-aconitate | 0.203 ± 0.08 | 0.188 ± 0.05 |
| creatinine | 5.431 ± 1.523 | 5.445 ± 1.249 |
| trimethylamine | 0.011 ± 0.00 | 0.011 ± 0.00 |
| methylguanidine | 0.046 ± 0.02 | 0.038 ± 0.03 |
| 5-aminolevulinate | 0.034 ± 0.01 | 0.043 ± 0.04 |
| dimethylamine | 0.158 ± 0.03 | 0.152 ± 0.03 |
| methylamine | 0.019 ± 0.01 | 0.015 ± 0.00 |
| citrate | 1.573 ± 0.78 | 1.339 ± 0.54 |
| succinate | 0.102 ± 0.08 | 0.091 ± 0.02 |
| pyroglutamate | 0.052 ± 0.10 | 0.052 ± 0.04 |
| pyruvate | 0.019 ± 0.01 | 0.036 ± 0.05 |
| 2-aminoadipate | 0.305 ± 0.15 | 0.315 ± 0.11 |
| acetone | 0.047 ± 0.25 | 0.009 ± 0.00 |
| methionine | 0.039 ± 0.04 | 0.030 ± 0.01 |
| acetate | 0.077 ± 0.04 | 0.122 ± 0.18 |
| lysine | 0.079 ± 0.04 | 0.081 ± 0.05 |
| alanine | 0.127 ± 0.05 | 0.140 ± 0.06 |
| dimethylmalonate | 0.005 ± 0.00 | 0.006 ± 0.00 |
| 2-phenylpropionate | 0.005 ± 0.00 | 0.008 ± 0.01 |
| 2-hydroxyisobutyrate | 0.03 ± 0.01 | 0.026 ± 0.01 |
| 3-hydroxyisovalerate | 0.038 ± 0.01 | 0.036 ± 0.01 |
| 3-aminoisobutyrate | 0.089 ± 0.13 | 0.058 ± 0.03 |
| propylene-glycol | 0.046 ± 0.04 | 0.042 ± 0.02 |
| methylsuccinate | 0.059 ± 0.04 | 0.078 ± 0.03 |
| 3-hydroxyisobutyrate | 0.047 ± 0.02 | 0.078 ± 0.12 |
| isoleucine | 0.005 ± 0.00 | 0.004 ± 0.00 |
| valine | 0.006 ± 0.00 | 0.005 ± 0.00 |
| 2-hydroxyvalerate | 0.016 ± 0.00 | 0.016 ± 0.00 |
| pantothenate | 0.014 ± 0.00 | 0.014 ± 0.00 |
| isocaproate | 0.030 ± 0.01 | 0.038 ± 0.02 |

**S1 Table**
