# Supplementary material for: Urine metabolome in women with Chlamydia trachomatis infection
Source: PLoS One. 2018 Mar 22;13(3):e0194827. doi: 10.1371/journal.pone.0194827 (PMC5864028; doi:10.1371/journal.pone.0194827)
Supplement: S2 Table — These data can be found on the ‘human metabolome database’ (HMDB; www.hmdb.ca), a freely available electronic database containing detailed information about small molecule metabolites found in the human body. (DOCX) [file pone.0194827.s003.docx]

| **Metabolite** | **References** |
| --- | --- |
| Hypoxanthine | - Boulieu R, Bory C, Baltassat P, Gonnet C. Hypoxanthine and xanthine levels determined by high-performance liquid chromatography in plasma, erythrocyte, and urine samples from healthy subjects: the problem of hypoxanthine level evolution as a function of time.  Anal Biochem 1983;129: 398-404.  - Boulieu R, Bory C, Baltassat P, Divry P. Hypoxanthine and xanthine concentrations determined by high performance liquid chromatography in biological fluids from patients with xanthinuria. Clin Chim Acta 1984; 142:83-89.  - Bouatra S, Aziat F, Mandal R, Guo AC, Wilson MR, Knox C, et al. The human urine metabolome. PLoS One 2013; 8:e73076. |
| 2-Furoylglycine | - Pettersen JE, Jellum E. The identification and metabolic origin of 2-furoylglycine and 2,5-furandicarboxylic acid in human urine. Clin Chim Acta 1972; 41: 199-207.  - Bouatra S, Aziat F, Mandal R, Guo AC, Wilson MR, Knox C, et al. The human urine metabolome. PLoS One 2013; 8:e73076. |
| Sucrose | - Tasevska N, Runswick SA, Welch AA, McTaggart A, Bingham SA. Urinary sugars biomarker relates better to extrinsic than to intrinsic sugars intake in a metabolic study with volunteers consuming their normal diet. Eur J Clin Nutr 2009; 63: 653-659.  - Tasevska N, Runswick SA, McTaggart A, Bingham SA. Urinary sucrose and fructose as biomarkers for sugar consumption. Cancer Epidemiol Biomarkers Prev 2005; 14:1287-1294.  - Bouatra S, Aziat F, Mandal R, Guo AC, Wilson MR, Knox C, et al. The human urine metabolome. PLoS One 2013; 8:e73076. |
| Threonine | - Cynober L, Blonde F, Nguyen Dinh F, Gerbet D, Giboudeau J. Measurement of plasma and urinary amino acids with gas chromatography in healthy subjects. Variations as a function of age and sex. Ann Biol Clin (Paris) 1983; 41:33-38.  - Bouatra S, Aziat F, Mandal R, Guo AC, Wilson MR, Knox C, et al. The human urine metabolome. PLoS One 2013; 8:e73076. |
| Lactate | - Bales JR, Higham DP, Howe I, Nicholson JK, Sadler PJ. Use of high-resolution proton nuclear magnetic resonance spectroscopy for rapid multi-component analysis of urine. Clin Chem. 1984 Mar;30(3):426-32  - Hušek P, Švagera Z, Hanzlíková D, Řimnáčová L, Zahradníčková H, Opekarová I, et al. Profiling of urinary amino-carboxylic metabolites by in-situ heptafluorobutyl chloroformate mediated sample preparation and gas chromatography-mass spectrometry. J Chromatogr A. 2016;1443:211-32.  - Bouatra S, Aziat F, Mandal R, Guo AC, Wilson MR, Knox C, et al. The human urine metabolome. PLoS One 2013; 8:e73076. |
| Mannitol | - Kubica P, Kot-Wasik A, Wasik A, Namieśnik J, Landowski P. Modern approach for determination of lactulose, mannitol and sucrose in human urine using HPLC-MS/MS for the studies of intestinal and upper digestive tract permeability. J Chromatogr B Analyt Technol Biomed Life Sci 2012; 907:34-40  - Bouatra S, Aziat F, Mandal R, Guo AC, Wilson MR, Knox C, et al. The human urine metabolome. PLoS One 2013; 8:e73076. |
| Methylguanidine | - Marescau B, Nagels G, Possemiers I, De Broe ME, Becaus I, Billiouw JM, et al. Guanidino compounds in serum and urine of nondialyzed patients with chronic renal insufficiency. Metabolism. 1997;46:1024-1031.  - Bouatra S, Aziat F, Mandal R, Guo AC, Wilson MR, Knox C, et al. The human urine metabolome. PLoS One 2013; 8:e73076. |
| Pyruvate | - Koike K, Koike M. Fluorescent analysis of alpha-keto acids in serum and urine by high-performance liquid chromatography. Anal Biochem 1984; 14:481-487.  - Hara S, Takemori Y, Yamaguchi M, Nakamura M, Ohkura Y. Determination of alpha-keto acids in serum and urine by high-performance liquid chromatography with fluorescence detection. J Chromatogr. 1985; 344:33-39.  - Bouatra S, Aziat F, Mandal R, Guo AC, Wilson MR, Knox C, et al. The human urine metabolome. PLoS One 2013; 8:e73076. |
| Acetone | - Fujino A, Satoh T, Takebayashi T, Nakashima H, Sakurai H, Higashi T, Matumura H, Minaguchi H, Kawai T. Biological monitoring of workers exposed to acetone in acetate fibre plants. Br J Ind Med 1992; 49:654-657.  - Bouatra S, Aziat F, Mandal R, Guo AC, Wilson MR, Knox C, et al. The human urine metabolome. PLoS One 2013; 8:e73076. |
| Methylsuccinate | - Hušek P, Švagera Z, Hanzlíková D, Řimnáčová L, Zahradníčková H, Opekarová I, et al. Profiling of urinary amino-carboxylic metabolites by in-situ heptafluorobutyl chloroformate mediated sample preparation and gas chromatography-mass spectrometry.  J Chromatogr A. 2016;1443:211-32.  - Suh JW, Lee SH, Chung BC. GC-MS determination of organic acids with solvent extraction after cation-exchange chromatography. Clin Chem. 1997;43:2256-61.  - Bouatra S, Aziat F, Mandal R, Guo AC, Wilson MR, Knox C, et al. The human urine metabolome. PLoS One 2013; 8:e73076. |

**S2 Table**
